# Long-term trends in cycle threshold values: a comprehensive analysis of COVID-19 dynamics, viral load, and reproduction number in South Korea

**DOI:** 10.3389/fpubh.2024.1394565

**Published:** 2024-08-12

**Authors:** Jungeun Park, Sung-il Cho, Sang-Gu Kang, Jee-Woun Kim, Sunkyung Jung, Sun-Hwa Lee, Kyou-Sup Han, Seung-sik Hwang

**Affiliations:** ^1^Graduate School of Public Health, Seoul National University, Seoul, Republic of Korea; ^2^Seegene Medical Foundation, Seoul, Republic of Korea

**Keywords:** cycle threshold value, COVID-19, reverse transcription polymerase chain reaction, testing, viral load

## Abstract

**Background:**

With the emergence of COVID-19 cases, governments quickly responded with aggressive testing, contact tracing, isolation and quarantine measures. South Korea’s testing strategy primarily relied on real-time reverse-transcriptase polymerase chain reaction (real-time RT-PCR), focusing on cycle threshold (Ct) values, indicative of viral load, to determine COVID-19 positivity. This study examined the long-term time series distribution of Ct values measured in the same laboratory using a nationally standardized testing type and sampling method in South Korea. It aimed to link Ct values, new COVID-19 cases, and the reproduction number (Rt), setting the stage for using Ct values effectively.

**Methods:**

This study analyzed nationally collected 296,347 samples Ct values from February 2020 to January 2022 and examined their associations with the number of new cases and Rt trends. The data were categorized into four COVID-19 periods for in-depth analysis. Statistical methods included time series trend analysis, local regression for smoothing, linear regression for association analysis, and calculation of correlation coefficients.

**Results:**

The median Ct values across four COVID-19 periods decreased gradually from 31.71 in the initial period to 21.27 in the fourth period, indicating higher viral load. The comparison of trends between Ct values and the number of new cases revealed that the decline in Ct values preceded the surge in new cases, particularly evident during the initial stages when new cases did not undergo a significant increase. Also, during variant emergence and vaccination rollout, marked shifts in Ct values were observed. Results from linear regression analysis revealed a significant negative relationship between Ct values and new cases (β = −0.33, *p* < 0.001, *R*^2^ = 0.67). This implies that as Ct values decrease, new case numbers increase.

**Conclusion:**

This study demonstrates the potential of Ct values as early indicators for predicting confirmed COVID-19 cases during the initial stages of the epidemic and suggests their relevance in large-scale epidemic monitoring, even when case numbers are similar.

## 1 Introduction

In South Korea, the first case of coronavirus disease 2019 (COVID-19) was confirmed on January 20, 2020 (1), and the government established its response measures based on aggressive testing, tracing, quarantine, and isolation (2, 3). Of these strategies, aggressive testing is at the core for preventing the spread of infection through the community by enabling early infection detection. To facilitate testing, the government launched multiple screening sites, implemented mobile sample collection teams and mobile health examination services, continually increased diagnostic testing counts, and added COVID-19 to the existing respiratory disease surveillance system (2). As a result, mass COVID-19 tests were done with quick turnaround times, and the government performed tracing, enforced quarantine, and provided treatment based on the test results. As such, South Korea was considered to have managed the COVID-19 pandemic well compared to other countries (4).

In South Korea, COVID-19 tests are primarily performed via the 2019 novel coronavirus real-time reverse-transcriptase polymerase chain reaction (real-time RT-PCR) test to detect viral genes, typically in upper and lower airway samples (5). The assay is designed to detect two or three regions of severe acute respiratory syndrome coronavirus 2 (SARS-CoV-2) genes that encode nucleocapsid (*N*), envelope (*E*), or spike (*S*) proteins or the RNA-dependent RNA polymerase (*RdRp*) gene (5). The cycle threshold (Ct) values, representing the amplification cycles needed to surpass a predetermined threshold, serve as a determinant for COVID-19 positivity (6). A sample is deemed positive when the Ct value of the target gene is equal to or smaller than the cutoff value (6). Because this value can also be understood as the viral load, a lower Ct value is interpreted as a higher viral load (7).

Many recent studies have utilized Ct values to establish associations between Ct values and demographic factors (8, 9). These studies have explored the correlation between Ct values and various factors such as the occurrence of confirmed cases (10), hospitalization and mortality rates (11, 12), identification of infection stages in patients (13, 14), prediction of clinical characteristics and disease severity (13, 15, 16), differences based on viral mutations and vaccination status (17–19), and clinical decisions regarding the need for isolation and quarantine (7). Moreover, a study has utilized Ct values in relation to non-pharmaceutical interventions (20).

Investigating transmission trends and predicting outbreak sizes during an epidemic provide valuable data for formulating effective response measures (21). Currently, the time-varying effective reproductive number (Rt) is employed as an indicator for predicting epidemic trends (22). However, Rt is contingent on the number of confirmed cases, and a significant limitation of this index is its inability to offer real-time predictions of epidemic size due to the delay between the latent period of a particular disease and the reporting of confirmed cases. To address this limitation, some studies have applied the modeling of infection rates and the utilization of surveillance system indicators (23–25) to large population distributions of Ct values, demonstrating the ability to predict epidemic trends. Additionally, others have incorporated Ct value distributions into existing Rt calculations to enhance the accuracy of predictions and compare their predictive capabilities (25).

This study examined the long-term time series distribution of Ct values measured in the same laboratory using a nationally standardized testing type and sampling method in South Korea. Furthermore, it sought to analyze the relationship between Ct values, new COVID-19 cases, and the reproduction number (Rt) through comparisons, thus establishing the basis for utilizing Ct values.

## 2 Materials and methods

### 2.1 Data

The RT-PCR test results used in this study were obtained from upper respiratory samples collected between February 2020 and January 2022 by the Korea Centers for Disease Control and Prevention. These samples were collected from various sources, including public health centers, temporary screening clinics, medical institutions’ screening clinics, and general medical clinics, spanning 14 out of 17 regions across South Korea.

The individuals tested comprised those exhibiting suspected COVID-19 symptoms, individuals with epidemiological links to confirmed cases, people aged 60 and above, as well as preemptive screening for infection-prone facilities. Positive cases from rapid antigen and emergency screening tests were also included.

Different assays were used in different periods. In the early stages, emergency use assays (February 7 to December 13, 2020: Allplex^™^ 2019-nCoV [SARS-CoV-2] Assay to detect the *E, RdRP*, and *N* gene targets) were used; the official assays (December 14, 2020, to January 31, 2022: Allplex^™^ SARS-CoV-2 Assay to detect the *E, RdRP/S*, and *N* gene targets) were used thereafter.

In this study, the total population of the sampling areas was 51,266,914, from which 37,253,185 samples were collected, of which Ct values were determined for 296,347 samples and found to be positive for the *RdRp/S* target. These positive cases represent 36.3% of all positive cases in South Korea.

The data concerning the number of newly confirmed COVID-19 cases were collected from January 20, 2020 (the date of the first confirmed case) up to January 2022. The number of newly confirmed cases encompasses domestically transmitted and imported cases, as reported in the daily COVID-19 report of the Integrated Management System for Epidemic Diseases provided by the Korea Disease Control and Prevention Agency. The study utilized the daily Rt data for South Korea, extracted from the COVID-19 dataset provided by “Our World In Data”^1^ a project of the Global Change Data Lab, a registered charity based in England and Wales. The estimation of Rt values was conducted using the Kalman filter, an established technique for Rt estimation. The specific Rt values employed in this study were derived from a research paper that applied the Kalman filter to estimate Rt for 124 countries, including South Korea (26).

### 2.2 Statistical analysis

In this study, we compared the time-series trends of daily median Ct values with daily the number of newly confirmed COVID-19 cases, and the Rt values. To compare the time-series trends, we employed local regression to smooth the data of Ct values, the number of newly confirmed COVID-19 cases, and Rt values. Local regression, also known as locally weighted scatterplot smoothing (LOWESS), is a non-parametric regression method used to estimate the underlying trend in a dataset by fitting multiple local regressions. Linear regression analysis was used for the correlation analysis. We introduced a time lag between Ct values and new cases and Rt for the association analysis to conduct a detailed analysis of the two indices. The analysis of time-series trends and correlations was conducted by comparing between the entire duration and four specific COVID-19 epidemic periods as delineated by the Korea Disease Control and Prevention Agency (27). The data were analyzed using the SAS 9.4 software (SAS Institute, Inc., Cary, NC, United States).

## 3 Results

Four COVID-19 periods occurred in South Korea between January 20, 2020, and January 31, 2022. We examined the distributions of daily Ct values, new cases, and Rt and analyzed their associations for the entire study period and the four COVID-19 periods.

**Figure 1:**
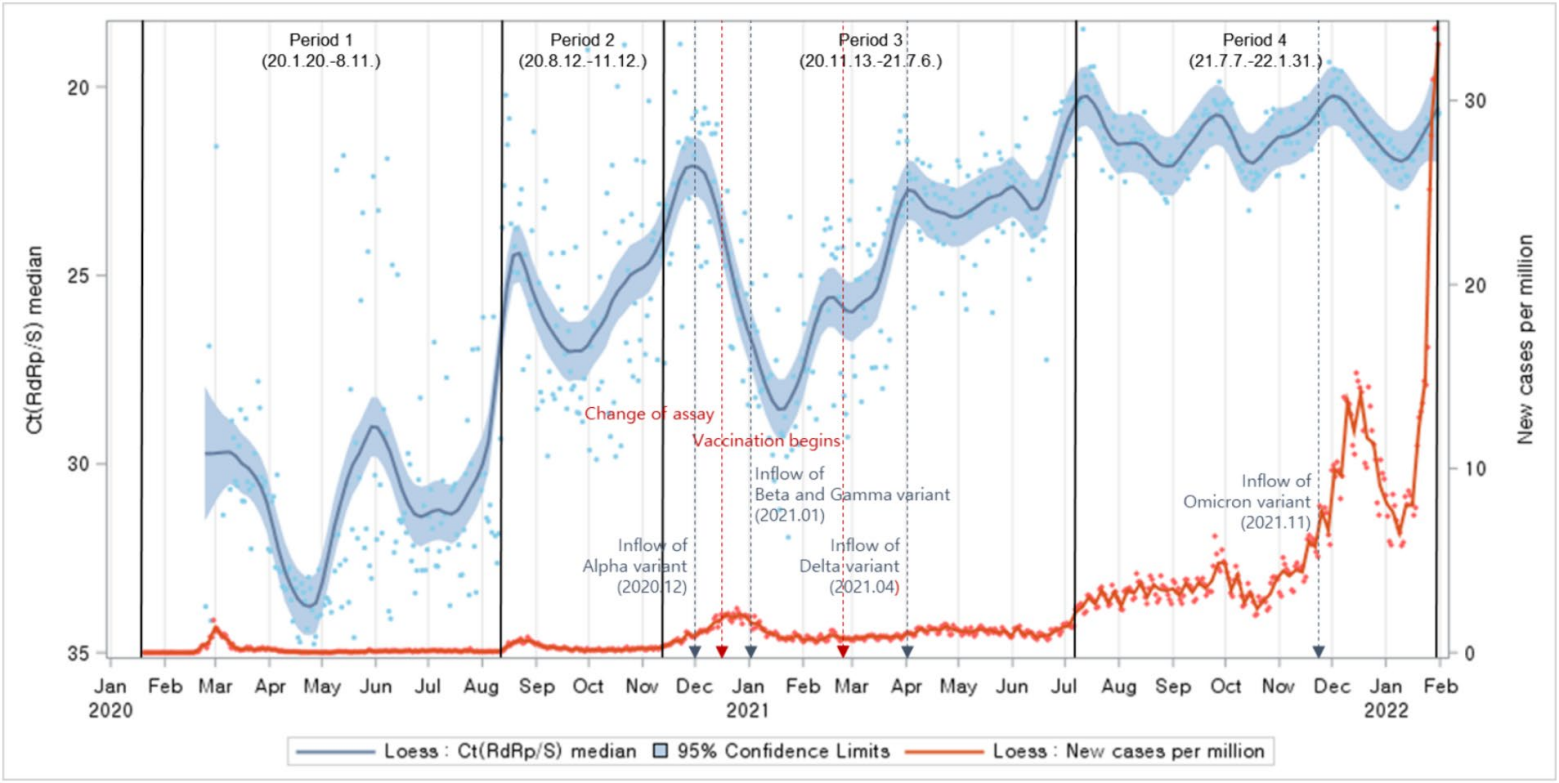
Distributions of Ct values and new cases per million population: evolution of the 1-day median Ct values (blue dot) and new cases per million (orange dot), obtained from February 2020, to January 2022 (Korea). Loess curves fitted to Ct median and new cases per million shown as a blue line (alpha = 0.75) and orange line (alpha = 0.75). Skyblue areas surrounding the blue line indicate the 95% confidence limits (CL) associated with median Ct values. The thick black vertical lines indicate the start and end of the four COVID-19 periods. The vertical red dashed lines denote changes in the testing assay and the commencement of vaccination begins. The vertical blue dashed lines indicate the onset of COVID-19 variants.

Ct values declined in general during the entire period, indicating an increased viral load over time. New cases surged after a substantial drop in the Ct values. Upon comparing the periods of reagent changes, vaccination campaigns, and the emergence of variant viruses with potential influences on Ct values, it became evident that Ct values were increasing even before the reagent change. After the vaccination campaigns, a slight decrease in Ct values was observed. In relation to the emergence of variant viruses, it was observed that Ct values exhibited an increasing trend following the emergence of the Alpha, Beta, and Gamma variants. After the emergence of the Delta variant, Ct values remained consistently low with no significant fluctuations, while following the emergence of the Omicron variant, Ct values exhibited an increasing trend (Figure 1).

**Figure 2:**
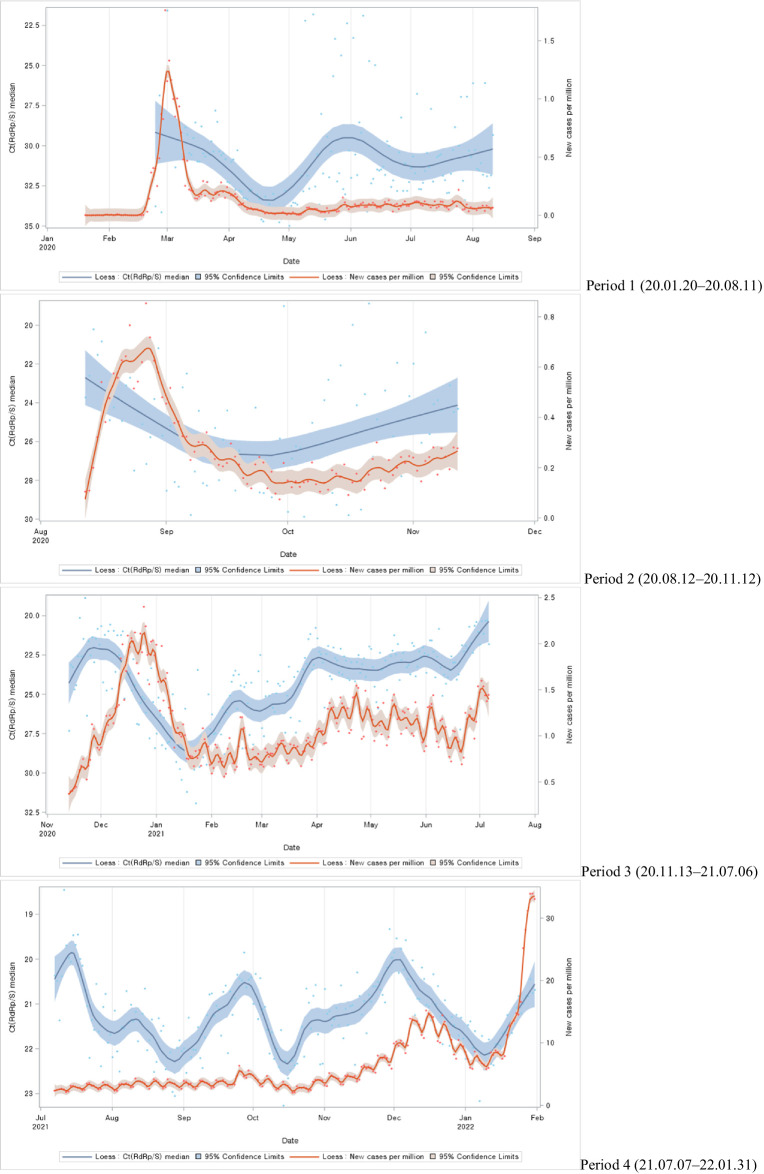
Distributions of Ct values and new cases per million during each COVID-19 period: evolution of the 1-day median Ct values (blue dot) and new cases per million (orange dot), obtained from February 2020, to January 2022 (Korea). Loess curves fitted to Ct median and new cases per million shown as a blue line (alpha = 0.75) and orange line (alpha = 0.75). Skyblue areas surrounding the blue line indicate the 95% confidence limits (CL) associated with median Ct values. Vivid orange areas surrounding the orange line indicate the 95% confidence limits (CL) associated with new cases per million.

The study categorized the time-series trends into different periods, each corresponding to different waves of the COVID-19 pandemic. In the first, second, and third periods, notable case spikes were primarily caused by cluster infections within specific regions. During these periods, a clear resemblance was observed between the patterns of Ct value trends and the trends of newly confirmed cases with a particular time lag. However, in the fourth period, characterized by large-scale outbreaks due to community transmission, although there were fluctuations in Ct values, no similarities were evident in the trends of newly confirmed cases (Figure 2).

**Figure 3:**
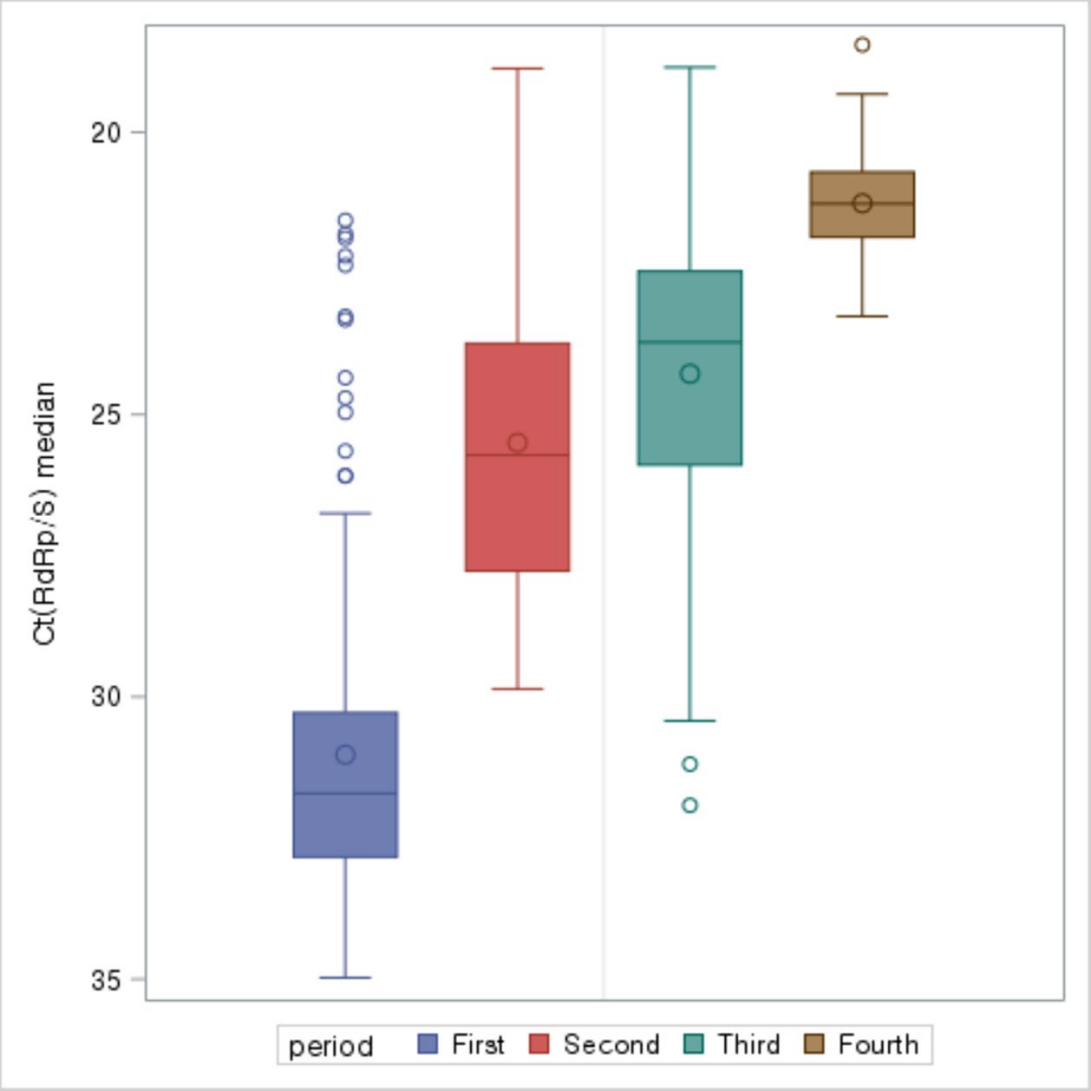
Ct values (median) by COVID-19 period.

The study compared the median overall Ct values for each period during the epidemic. The median Ct value was highest (31.71) in the first period and decreased over time, with the lowest value (21.27) in the fourth period (Figure 3).

**Figure 4:**
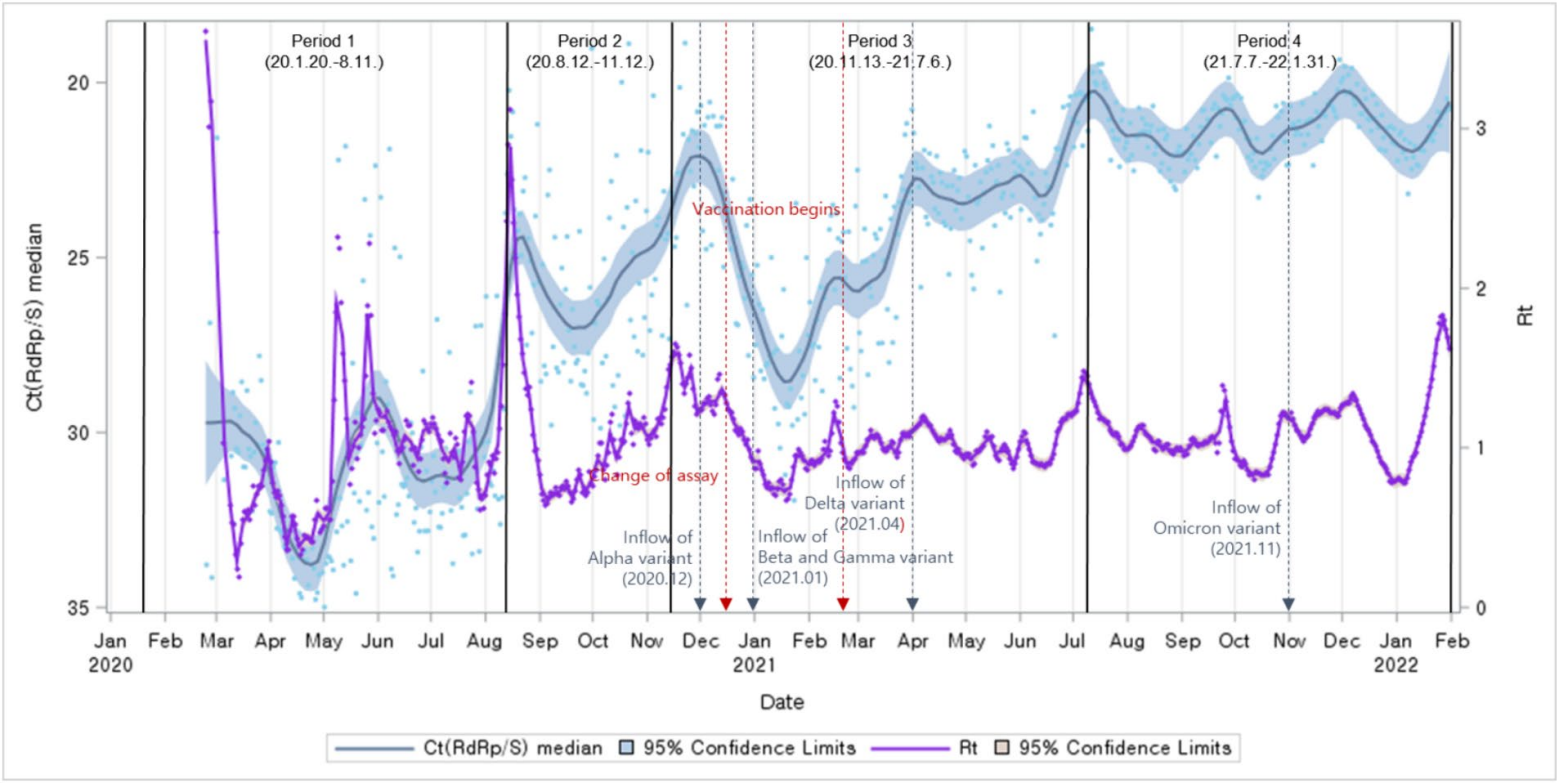
Distributions of Ct values and Rt: evolution of the 1-day median Ct values (blue dot) and Rt (purple dot), obtained from February 2020, to January 2022 (Korea). Loess curves fitted to Ct median and Rt shown as a blue line (alpha = 0.75) and purple line (alpha = 0.75). Skyblue areas surrounding the blue line indicate the 95% confidence limits (CL) associated with median Ct values. The thick black vertical lines indicate the start and end of the four COVID-19 periods. Vivid orange areas surrounding purple line indicate the 95% confidence limits (CL) associated with Rt.The vertical red dashed lines denote changes in the testing assay and the commencement of vaccination begins. The vertical blue dashed lines indicate the onset of COVID-19 variants.

Next, we compared Ct value trends with Rt. The Ct values trend revealed a pattern similar to the Rt fluctuations trend (Figure 4).

**Figure 5:**
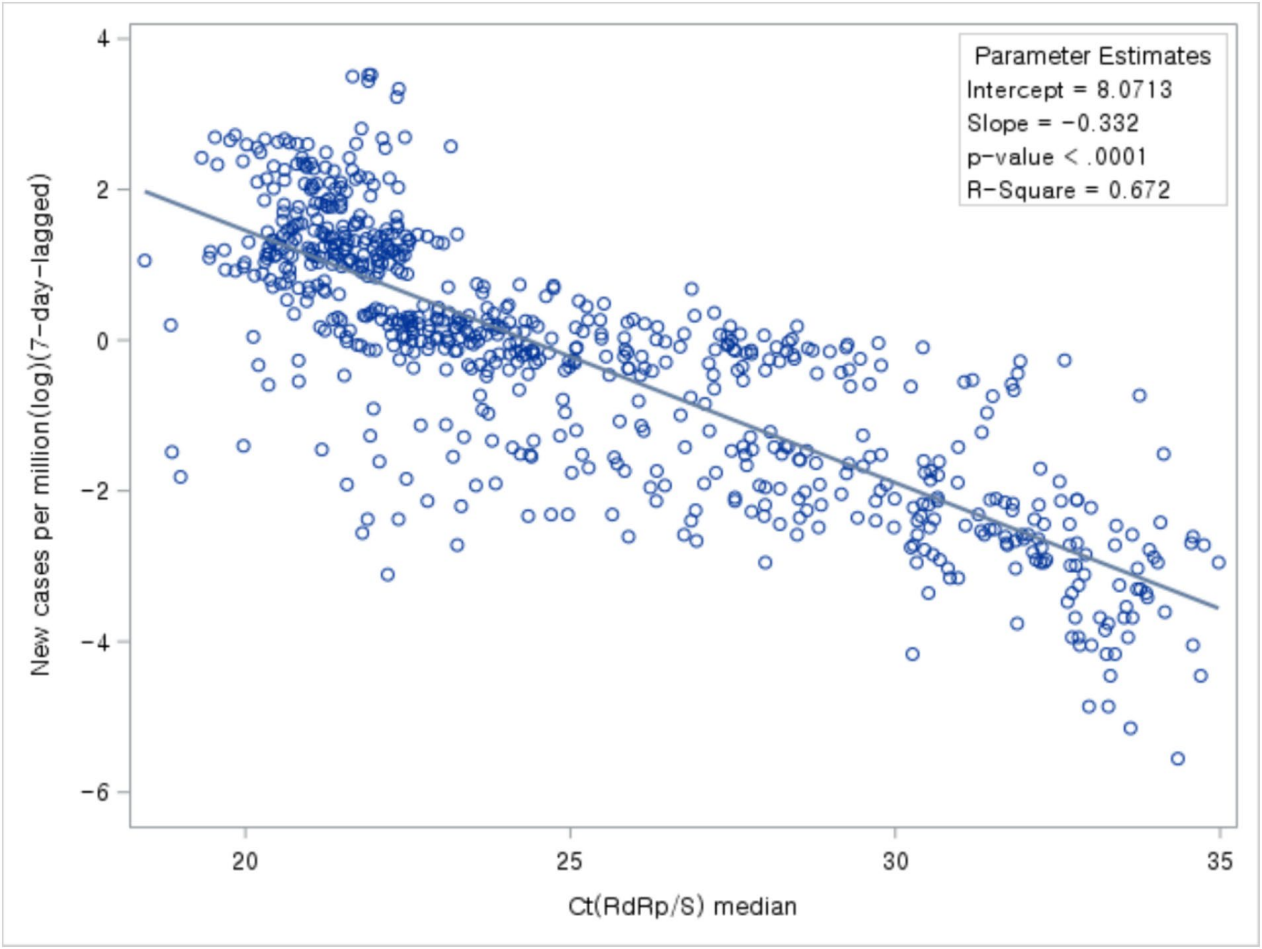
Association between Ct values and number of new cases.

The study analyzed the relationship between Ct values, the number of newly confirmed cases, and Rt. While comparing Ct value trends with the occurrence of newly confirmed cases and Rt trends, a time lag was observed. Based on the correlation analysis between the daily median Ct values and the occurrence of newly confirmed cases, the highest correlation coefficient between the two variables was observed with a time lag of 7 days. Notably, Ct values and the number of newly confirmed cases occurring 7 days later exhibited a negative correlation (Figure 5).

**Figure 6:**
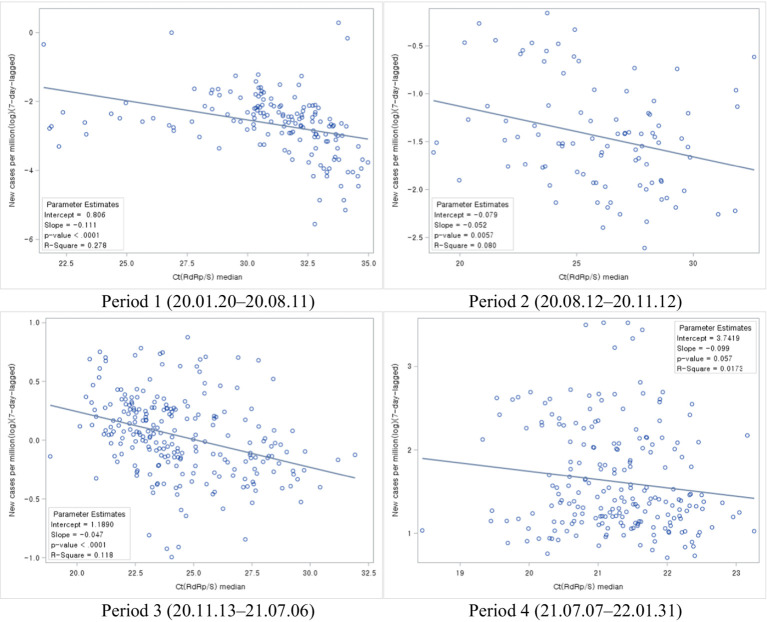
Association between Ct values and the number of new cases during each COVID-19 period.

Next, we analyzed the association between Ct values and new cases per COVID-19 period. During all four periods, new cases declined with increasing Ct values (Figure 6).

**Figure 7:**
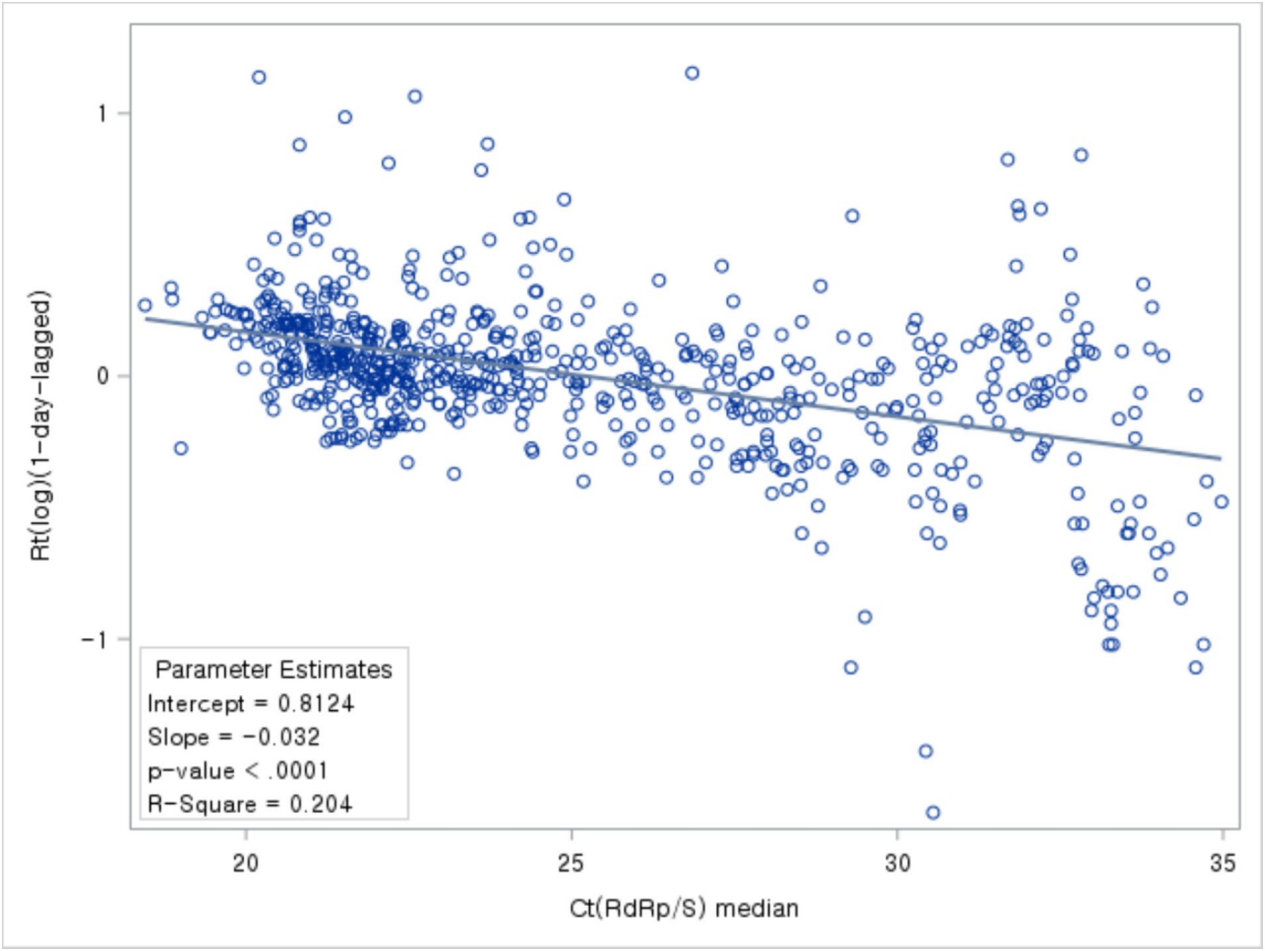
Association between Ct values and Rt.

Based on the correlation analysis between the daily median Ct values and Rt, the highest correlation coefficient between the two variables was observed with a time lag of 1 day. Notably, the analysis of the relationship between Ct values and Rt 1 day later exhibited a negative correlation (Figure 7).

**Figure 8:**
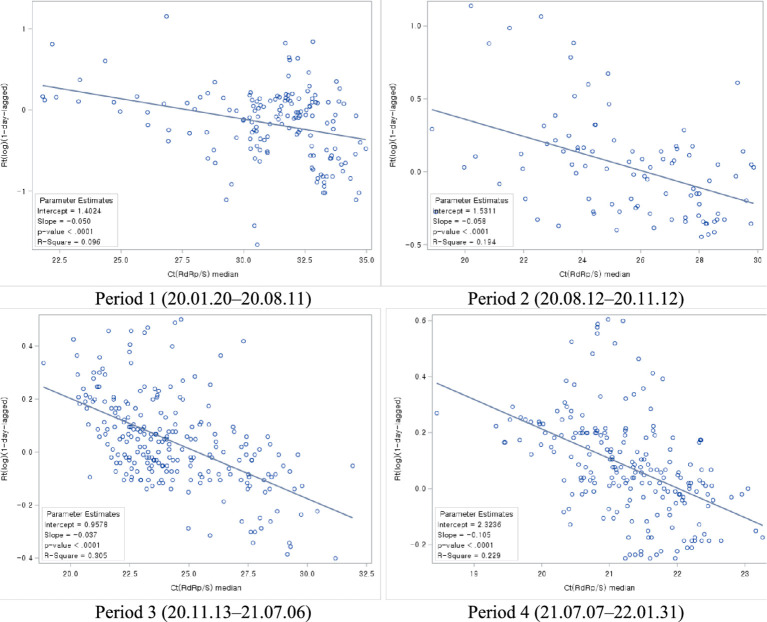
Association between Ct values and Rt during each COVID-19 period.

In conclusion, the analysis of the relationship between Ct values and Rt, divided by epidemic periods, revealed a consistent pattern. In all periods, it was observed that as Ct values increased, Rt decreased, indicating a negative correlation between the two variables (Figure 8).

## 4 Discussion

Since the first case of COVID-19 in the country on January 20, 2020, South Korea established an aggressive screening testing strategy to ensure early detection of individuals who developed the infection. Patients were traced, quarantined, and treated as necessary. Based on these implemented strategies, South Korea is considered to have done well in COVID-19 management compared to other countries. COVID-19 testing was performed via real-time RT-PCR, with Ct values, and the number of amplification cycles needed to cross a certain threshold, used to determine positivity. This value can also be understood as corresponding to the patient’s viral load.

Recently, there has been a shift away from the binary use of Ct values for determining COVID-19 positivity toward research that explores their utilization in understanding demographic variations, correlations with hospitalization and mortality rates, identification of infection stages in patients, and their relevance to non-pharmaceutical interventions (8–20). Moreover, some studies have delved into applying Ct values to predict future epidemic trends and monitor infectious diseases (23–25).

In this current study, we sought to examine the long-term time series distribution of Ct values measured in the same laboratory using nationally standardized testing types and sampling methods in South Korea. Additionally, we aimed to contribute to the establishment of the basis for utilizing Ct values by analyzing their relationship through comparisons with new COVID-19 cases and the Rt.

From January 20, 2020, to January 31, 2022, there were four COVID-19 periods in Korea. Here, we analyzed the associations between daily Ct values, the number of new cases, and the Rt for the entire COVID-19 period and each COVID-19 period. Ct values generally decreased during the entire period analyzed, indicating an increased viral load over time. We also observed that new cases surged after a substantial drop in Ct values. Regarding the four different COVID-19 periods, the average daily number of new cases was markedly higher during the second period (142.8) than during the first period (71.5), even though the cumulative number of cases was similar between the two periods, showing that the virus spreads more rapidly when Ct values are low.

The trajectory of Ct values displayed a resemblance to the trend observed in Rt, a predictive indicator of incidence. Analysis of their association with new cases, incorporating time lag, revealed that Ct values anticipate the onset of cases marginally sooner than Rt.

Exploring the factors influencing Ct values, notably variant emergence and vaccination endeavors, delineated distinct shifts pre- and post-events. The ascendancy of the Alpha variant in December 2020 marked the onset of variant dominance, persisting until mid-July. The subsequent emergence of the Beta and Gamma variants in January 2021 coincided with an observable uptrend in Ct values. The Delta variant’s dominance from April 2021 to December 2021 ushered in a phase of consistently low Ct values devoid of significant fluctuations. However, the emergence of the Omicron variant in late November 2021 heralded a noticeable uptick in Ct values. Further analysis underscored a decline in Ct values following the inception of vaccination efforts.

Several limitations need to be acknowledged in our study. Firstly, while our study data were collected at the national level, they only represented 40% of the total data, with test results missing for two out of 17 cities and provinces in Korea. This incomplete coverage may introduce potential biases.

Furthermore, there are inherent limitations associated with utilizing Ct values and confirmed case occurrences as supplementary variables. The lack of precise timing for virus infection and sampling during specimen collection for Ct value determination introduces uncertainty. This uncertainty regarding the exact timing of virus infection and sampling points may hinder our ability to fully and accurately account for differences in Ct values and the occurrence of confirmed cases.

In addition, our use of daily median values for Ct values has its own limitations. This approach does not consider the sampling variability inherent in the median itself nor explores various trends and analyses that could account for factors like data skewness.

Despite these limitations, our study’s data, collected at the national level of viral load, holds potential applicability in diverse settings, especially within the context of surveillance. This dataset could prove valuable in nationwide applications, including cluster epidemiological investigations and implementing COVID-19 control measures such as testing, contact tracing, and isolation/quarantine at the national level.

Moreover, there is a pressing need for comparative studies examining the impact of non-pharmaceutical interventions, such as mask-wearing and social distancing, on Ct values, the incidence of newly confirmed cases, and the effective Rt, depending on the timing of their implementation.

Finally, the significant association between Ct values and COVID-19 case occurrence 1 week later, as revealed by our study, underscores the importance of adjusting for potential confounding effects of confirmed cases from 1 week prior when analyzing these variables. Such adjustments will facilitate a clearer understanding of the relationship between Ct values and confirmed cases and highlight their utility as predictive factors.

As observed in this study, the negative correlation between Ct values and the number of new cases in the overall population is consistent with previous reports (11, 24, 28). Similar correlations were observed in studies focusing on hospital settings and local populations (29, 30). Furthermore, Avadhanula et al. (29) found that the correlation between Ct values and the number of new cases after 1 week was strongest among the factors studied.

In this study, by analyzing the trends of Ct values measured in the same laboratory using nationally standardized testing types and sampling methods, we were able to confirm the potential utility of Ct values for predicting the occurrence of confirmed cases during the early stages of the epidemic when the number of cases is low. Furthermore, even when the scale of confirmed cases is similar, we discovered variations in the extent of virus spread based on Ct values. As suggested by Yin et al. (31) and Musalkova et al. (32), this study also proposes the utilization of large-scale epidemic and infectious disease monitoring incorporating Ct value information.

## Data availability statement

The datasets presented in this article are not readily available because the access rights to some of the datasets used in this study are owned by “Seegene Medical Foundation.” Requests to access the datasets should be directed to SL, lshkim@mf.seegene.com.

## Ethics statement

The studies involving humans were approved by the Institutional Review Board (IRB) of Seoul National University (IRB No. E2207/001-002). The studies were conducted in accordance with the local legislation and institutional requirements. The ethics committee/institutional review board waived the requirement of written informed consent for participation from the participants or the participants’ legal guardians/next of kin because The institution providing the data has already submitted approved IRB documents.

## Author contributions

JP: Writing – original draft, Writing – review & editing. SC: Writing – original draft, Writing – review & editing. S-GK: Writing – review & editing. J-WK: Writing – review & editing. SJ: Writing – review & editing. SL: Writing – review & editing. K-SH: Writing – review & editing. SH: Writing – original draft, Writing – review & editing.

## Footnotes

^1^ https://ourworldindata.org/coronavirus
